# Complications and discomfort of bronchoscopy: a systematic review

**DOI:** 10.3402/ecrj.v3.33324

**Published:** 2016-11-11

**Authors:** Elise Orvedal Leiten, Einar Marius Hjellestad Martinsen, Per Sigvald Bakke, Tomas Mikal Lind Eagan, Rune Grønseth

**Affiliations:** 1 Department of Clinical Science, University of Bergen, Bergen, Norway; 2 Department of Thoracic Medicine, Haukeland University Hospital, Bergen, Norway

**Keywords:** diagnostic bronchoscopy, safety, adverse events, patient satisfaction, informed consent

## Abstract

**Objective:**

To identify bronchoscopy-related complications and discomfort, meaningful complication rates, and predictors.

**Method:**

We conducted a systematic literature search in PubMed on 8 February 2016, using a search strategy including the PICO model, on complications and discomfort related to bronchoscopy and related sampling techniques.

**Results:**

The search yielded 1,707 hits, of which 45 publications were eligible for full review. Rates of mortality and severe complications were low. Other complications, for instance, hypoxaemia, bleeding, pneumothorax, and fever, were usually not related to patient characteristics or aspects of the procedure, and complication rates showed considerable ranges. Measures of patient discomfort differed considerably, and results were difficult to compare between different study populations.

**Conclusion:**

More research on safety aspects of bronchoscopy is needed to conclude on complication rates and patient- and procedure-related predictors of complications and discomfort.

Flexible bronchoscopy (FB) was introduced in 1968, and today it is an essential procedure in respiratory medicine. There are numerous indications for bronchoscopy, and it is frequently used for diagnostic and therapeutic purposes in both inpatients and outpatients. White light FB is commonly used in diagnostics, as it enables visualisation of the lower airways and sampling techniques such as bronchial brushings (BB), bronchial washings (BW), bronchoalveolar lavage (BAL), endobronchial biopsies (EBB), transbronchial biopsies (TBB), and transbronchial needle aspiration (TBNA) (1).

Bronchoscopy is generally considered safe (2). However, whether performed with anaesthesia or only light sedation, pre-procedural medications are routinely administered and may have side effects. Diagnostic sampling may lead to immediate, although rare, complications, such as intrabronchial bleeding, bronchospasm, and pneumothorax. In addition, some discomfort may be felt in the days after the procedure, such as fever, sore throat, cough, or reactions to the medications used (1). Events occurring after the observation period may not be detected by the bronchoscopist. To ensure that both the bronchoscopy team and the patient are adequately prepared for the procedure, a realistic picture of the potential for complications and discomfort is imperative.

To the best of our knowledge, there is no recent systematic review of complications and discomfort associated with bronchoscopy. The 2013 British Thoracic Society Guidelines (2) includes a comprehensive overview of complications, but only presents a few selected references without discussing potential weaknesses of the included studies.

Thus, we set out to conduct a systematic review of complications and patient discomfort associated with non-interventional bronchoscopy, and the frequency and predictors of these in patients and research subjects.

## Methods

We used a modified Population - Intervention - Outcome comparison (PICO) form (3) (Table 1) and performed a systematic literature search in PubMed (Medline). Keywords were selected by combining existing thesauruses (MeSH terms) and text words. We performed a review of the existing MeSH database and of the (MeSH) classification of relevant papers that were already published. In addition, we added text words considered relevant to describe complications known to the authors.

**Table 1:** Search word combinations, in a modified PICO form for a systematic literature search on complications and discomfort related to bronchoscopy

We are interested in a procedure called (Intervention 1)	Where … is performed. (Intervention 2)	Will it lead to …? (Outcome)
Bronchoscopy	Bronchoalveolar lavage	Complication^a^
	BAL	Discomfort
	Brush^a^	Cough^a^
	Transbronchial biopsy	Saturation decrease
	Endobronchial biopsy	Adverse events
	Bronchial biopsy	Adverse effects
	Conscious sedation	Bronchospasm
	Lidocaine	Death
		Pneumothorax
		Shortness of breath
		Dyspnoea
		Bleeding
		Haemorrhage
		Fever
		Vasovagal syncope
		Cardiac arrest
		Contraindication
		Safety
		Patient experience
		Adverse symptoms
		Anxiety
		Pain
		Hospitalisation

a Truncation. The content of columns was combined with OR. Different columns were combined with AND.

The search in PubMed was conducted on 8 February 2016.

We included publications in English, Norwegian, Swedish, Danish, and French. Case reports, non-original research (letters, review articles, guidelines, etc.), animal studies, studies solely based on interventional procedures and specialised examination techniques, studies on paediatric populations as well as studies of intubated patients, patients on mechanical ventilation, under general anaesthesia or in an intensive care unit (ICU), were excluded, along with publications that did not cover the topic on complications or discomfort associated with bronchoscopy. Studies on bronchoscopes as a source of contamination were considered outside the scope of the current review.

Papers were classified as prospective or retrospective, and whether investigation of complications and discomfort was considered an objective (primary, secondary, not formalised). We also divided articles into three groups based on the number of subjects in the study and identified studies on medication during or before bronchoscopy.

Full review was only performed on papers where complications or discomfort was a primary or secondary objective of the study, where the number of subjects exceeded 50, and where there was given a sufficient description of the sample and the sampling methods (inclusion/exclusion criteria, definition of endpoints, and data collection). We chose to exclude papers based on less than 50 subjects since the statistical power of these studies in detecting rare complications is bound to be low.

## Results

The initial literature search yielded 1,707 papers, of which 1,435 were excluded (Table 2). In total, 94 papers reported complications and discomfort as their primary or secondary objective in procedures on more than 50 subjects (Table 3). Of these papers, 15 did not define outcomes sufficiently (4–18), five papers did not give information on the data collection (6, 7, 13, 14, 19), four papers were based on surveys of health care suppliers (20–23), and inclusion or exclusion criteria were not specified in 37 papers (8, 9, 12, 17, 20–52). Thus, further review was performed on the remaining 45 publications. The articles are subsequently reviewed with respect to the subtopics: death, bleeding, pneumothorax, bronchospasm, hypoxaemia, haemodynamic variations, fever and infection, health care utilisation, coughing, other respiratory symptoms and signs, and identified discomfort and pain The publications are further described in the Supplementary file.

**Table 2:** Yield of a PubMed – literature search on discomfort and complications related to bronchoscopy (8 February 2016)

	Number of articles
Total in search	1,707
Type of publications	
Excluded, non-original	214
Excluded, language	183
Excluded, case studies	268
Excluded, not human	37
Type of bronchoscopy	
Excluded, provocation test	24
Excluded, interventional bronchoscopy	26
Excluded, general anaesthesia/intubated/ mechanical ventilation/ICU	149
Excluded, endobronchial ultrasound (EBUS)	32
Excluded, experimental or non-standard bronchoscopy techniques	7
General	
Excluded, no relevance/does not address complications nor patient experience	381
Excluded, children	110
Excluded, disease outbreak study	3
Excluded, did not report according to objective^a^	1
Publications excluded, total	1,435
Publications remaining, total	272

a One study did not report complications, despite the objective ‘(…) to document any complications’.

**Table 3:** Quantitative overview of articles from a systematic literature search on complications and discomfort of bronchoscopy, divided into groups based on study design characteristics, number of subjects investigated, and relevance to the topic of complications and discomfort

	Subjects	Primary objective	Secondary objective	Reports complication	Claims ‘no complications’	Total
Prospective studies	*n*>200	14^a^	3^a^	4	0	21
	*n* 50–200	31^a^	2^a^	26	4	63
	*n*<50	28	4	31	11	74
Retrospective studies	*n*>200	15^a^	4^a^	12	3	34
	*n* 50–200	3^a^	2^a^	13	2	20
	*n*<50	8	7	7	7	29
Medication studies	*n*>200	5^a^	0	0	0	5
	*n* 50–200	15^a^	0	0	0	15
	*n*<50	8	0	3	0	11
Total		127	22	96	27	272

a In total, 94 articles reported complications and discomfort as their primary or secondary objective in procedures on more than 50 subjects.

### Death

Nine papers specified death as a potential outcome (53–61). The studies comprised 71–702 subjects (53, 61). All studies, except Grendelmeier et al. (59, 61), were conducted on selected populations (mostly immunocompromised individuals). As in all but one study (53), Grendelmeier et al. report a mortality rate of 0% (59, 61).

### Bleeding

Bleeding rates varied between 2.5 and 89.9% in the prospective studies and drug studies (59, 62). The studies comprised 88–1,217 subjects (63, 64). Some studies graded severity of bleeding according to volume (58, 62, 65–67), whereas others graded in terms of required intervention (63, 64, 68–70). Three studies did not define bleeding (59, 61, 71). Carr et al. aimed to investigate actual blood loss in 234 patients with low risk of bleeding. They categorised bleeding as minimal (<5 ml), mild (5–20 ml), moderate (20–100 ml), and severe (>100 ml) and found that 89.7% had minimal bleeding, 8.1% had mild bleeding, and 2.1% had moderate bleeding. No patients had severe bleeding. Superior vena cava syndrome and addition of EBB and TBB to TBNA predicted bleeding (62).

### Pneumothorax

Six prospective studies (57, 58, 64–66, 72) and two retrospective studies (55, 73) listed pneumothorax as a potential outcome, with rates ranging from 0 to 4% (58, 72). Two studies reported no pneumothoraces in various bioptic techniques that included TBB (66, 72). Jain et al. reported 4% pneumothorax but did not relate complications to the specific procedure (58). Dang et al. reported that pneumothorax occurred in three patients at a rate of 1.6% when expressed as a percentage of TBB. One pneumothorax required intervention (57). Herth et al. conducted a study on 1,217 patients going through TBB and found that 26 of them (2.1%) developed pneumothoraces, of which 14 were treated with tube thoracostomy, and the remaining 12 required no intervention (64). There were no prospective studies reporting pneumothorax as a result of other sampling procedures, such as brush sampling or lavage.

A large, retrospective population-based register study found that 0.97% (95% confidence interval (CI): 0.94–1.01%) of transbronchial lung biopsies were complicated by a pneumothorax that required chest tube placement (73).

### Bronchospasm

Three prospective studies (57, 74, 75) and one retrospective study with prospective recordings of bronchospasm (55) reported on bronchospasm. Bronchospasm occurred at a rate between 0 and 12.3% (57, 75). The rate of 12.3% was found in a study including asthma patients exclusively (75).

### Hypoxaemia

Ten studies provided information on hypoxaemia in unselected, elective patients (59, 61, 63, 76–82). The studies comprised 73–702 subjects (61, 79). The majority of these prospective studies and drug studies defined hypoxaemia, or desaturation, as an oxygen saturation ≤90% (59, 61, 63, 76–80, 82) or as a drop in pO_2_ to <60 mmHg at varying time points (81). The papers reported desaturation rates between 0.7 and 76.3% (80, 81). Rates around 75.0% (duration not defined) were observed in both subjects with (78) and without supplemental oxygen (77, 81). Fruchter et al. aimed at conscious sedation (propofol), which is defined as being able to rouse the patient by mild prodding or shaking (78). Grendelmeier et al. reported more mid-range results, with desaturation less than 90% in 16.4% of 440 patients going through bronchoscopy with propofol sedation, with conscious sedation defined as onset ptosis (59). Of note is that only two out of 10 studies specified a level of hypoxaemia at which they considered bronchoscopy contra-indicated (77, 79).

### Haemodynamic variations

Eight drug studies (61, 70, 74, 76, 78, 79, 82, 83) and two prospective studies (59, 63) reported haemodynamic complications. The studies comprised 72–702 subjects (61, 79). In six papers, hypotension was regarded as a systolic blood pressure (SBP) of <90 mmHg (59, 61, 63, 76, 78, 79) that required intervention (83). Hypotension ranged from 2.9%, in patients sedated with propofol and dexmedetomidine (79), to 28.9% in propofol sedation (61). Two papers reported that 1–16% of participants needed fluid resuscitation due to hypotension (78, 83). No paper reported clinical outcome associated with hypotension. Only two studies defined hypertension: one as SBP>180 mmHg or diastolic BP>90 mmHg (63) and the other as BP>140/90 (79). Bradycardia was defined in three studies, <60/min (79), <55/min (70), and <50/min, and required intervention (83). All reported the incidence of bradycardia to be 0. Two drug studies defined tachycardia, >100/min (79) and >130/min (70), and reported incidence rates of 25.7% (79) and 8.0% (70), respectively. Ryu reported 10.0% arrhythmias (79). Information regarding the need of anti-arrhythmic therapy was not given in any of these studies (70, 79).

### Fever and infection

Elevation of body temperature was reported in seven prospective studies (57, 65, 77, 84–87) and one retrospective study with prospective recordings of temperature (55). The studies comprised 50–539 subjects (57, 85). The range in incidence was 2–33% (77, 84). No studies used comparable definitions of ‘fever’ or ‘temperature change’. Krause et al. defined fever as a rise in body temperature to >38°C. Axillary body temperature was measured in the morning prior to bronchoscopy and 3, 6, 12, and 24 h after examination. In 20 patients, BAL was performed; 30 patients were examined by bronchoscopy only; 12 patients (24%) developed fever. There was no difference between the BAL and non-BAL groups (85). González Aguirre et al. reported an increased symptomatology in 65.1% post-FB and stated that this was mainly due to fever. The number of patients experiencing fever was not reported (87). Other signs, symptoms, and findings related to infection were reported in six prospective studies (75, 77, 85–88). Yigla et al. studied 200 patients without pre-procedural pulmonary infection and found a 6.5% of bacteraemia rate following bronchoscopy (88). In a study of asthma patients, 7% experienced respiratory infection during the 2 weeks following bronchoscopy, but antibiotic treatment or other required intervention was not reported (75). Krause et al. found flu-like symptoms in 8 out of 12 patients with fever, and two with chills and severe constitutional symptoms, all of whom responded well to Non Steroid Antiinflammatory Drugs (NSAIDs) and subsided within 24 h (85). Pereira et al. reported that one patient with protracted fever had a progressive pneumonitis with a fatal outcome following bronchoscopy despite antimicrobial drug therapy. All other cases of fever subsided without antimicrobial treatment (86).

### Health care utilisation

Nine prospective studies (57–59, 61, 65, 68, 70, 84, 86) reported complications that had to be handled by increased health care utilisation. Similarly, five retrospective studies (53–56, 73) reported events of increased healthcare utilisation. Tukey and Wiener used health care registers to identify pneumothoraces and haemorrhages coded as iatrogenic and subsequently attributed them to bronchoscopic procedures (73). The remaining studies comprised 71–702 subjects (53, 61). The incidence of health care contacts ranged from 0 to 31%, (59, 60) but was difficult to compare across different studies and designs. We were not able to conclude regarding admission rates, prolonged observation after bronchoscopy, or regarding assistance from outpatient/emergency room services after the initial in-hospital observation.

### Coughing

In some papers, coughing was referred to as a complication or adverse event (57, 59, 63, 65, 71, 75, 87, 89), and in others it was simply a measure of discomfort (61, 72, 74, 82, 90, 91). Six prospective studies, comprising 57–539 subjects (57, 75), reported cough by giving the proportion of patients who experienced or were bothered by coughing (57, 63, 65, 71, 72, 75). In these studies, the rate ranged from 4.7 to 86.0% (65, 72). Procedural cough was investigated in five articles (63, 65, 71, 72, 75). Post-procedural cough was investigated in two papers, with an incidence of 10.8% (57) and 55.7% (63). None of the above-mentioned papers reported on the duration of cough. Visual analogue scale (VAS), numeric rating scale (NRS), and cough counting were the main rating tools of cough in the drug trials; however, results were difficult to compare as they investigated different drug regimens and primarily reported differences in cough related to sedation or topical anaesthesia in subgroups within the trial.

### Other respiratory symptoms and signs

Papers reporting on respiratory symptoms besides cough and bronchospasm included five prospective studies (57, 61, 71, 75, 92). Two papers investigated change in asthma symptom scores in a 2-week period following bronchoscopy. Humbert et al. found no change in asthma score (92), whereas Tapanainen et al. found that 5.3% had an increase in asthma symptoms (75). Two papers reported rates of dyspnoea between 3.5% (75) and 5.7% (57) as observed by the researchers. In a study by Choi et al., self-reported shortness of breath was 38.2 and 30.9% in subgroups of nasal and oral insertion of bronchoscope, respectively (71). In other studies investigating patient-reported dyspnoea, rates were not possible to extract as only the ratios between subgroups were given in the papers (53–59, 61, 65, 68, 70, 73, 84, 86).

### Identified discomfort and pain

Eight prospective studies (63, 71, 72, 77, 87, 89, 93, 94) and 12 drug studies (60, 61, 74, 76, 79, 82, 83, 90, 91, 95–97) reported subjective measures of patient satisfaction or discomfort related to bronchoscopy. Numeric rating scales (NRS), verbal analogue scales (VAS) and visual analogue scales (VAS) were the most common assesment tools. Several different scales were employed: verbal analogue scales from 0 to 10 (63, 76): 10-point Likert scale (1–10, 83); VAS 0–10 cm (with opposite orientations) (60, 82, 87, 90, 91, 96, 97); VAS 0–100 mm (with opposite orientation) (72, 89, 95); NRS 0–100 (79); faces pain rating scale (0–5) (74); and grading distress as no, some, or extreme distress (77). Drug studies and studies evaluating different clinical interventions used these scales to compare the patient satisfaction between the intervention groups (60, 63, 74, 76, 79, 82, 83, 87, 89–91, 94–97). The only measure of satisfaction that was comparable between studies was ‘willingness to return’, which was used in six studies (61, 63, 71, 72, 79, 83) ranging between 55.4 and 96.3% (61, 71).

## Discussion

We have presented a systematic review on complications and discomfort of FB. Severe complications were rare; pneumothorax requiring intervention was reported in 0–2.1% of patients who had undergone TBB (64, 72). Mortality rate was low, but it was difficult to compare between studies that were performed on more or less selected populations. The willingness to repeat bronchoscopy was well above 50%.

Rates of specific complications ranged considerably, as in the case of oxygen desaturation [0.7–76.3% of patients (80, 81)] and bleeding (2.5–89.9% of patients) (59, 62). There are several potential reasons for this: the wide range of definitions (discussed below), different schemes for data collection, differences in equipment and techniques, differences between patient populations, and possibly time-dependent inter-study differences, as there are more than 40 years of gap between the publications included in this review. We argue that the considerable variability in complication rates can be attributed to a lack of consensus on how to define and measure complications and that many of the presented studies have a modest sample size.

Patient tolerance was difficult to assess as all studies utilised different measures of discomfort. VAS and NRS were mostly used to compare subgroups receiving different drug regimens, and it was unclear whether the results of these studies were representative for clinical practice.

Furthermore, absolute scale values were rarely presented in result sections, as relative comparison between subgroups was preferred.

The closest we got to a mortality rate that is representative of routine clinical practice was in one of the excluded studies. Facciolongo and co-workers reported a mortality rate of 0.02% in a large prospective study in 19 centres conducting diagnostic and therapeutic bronchoscopy. All deaths were somehow related to patients with a scheduled bronchoscopic laser treatment. This report was excluded from our main review because the authors did not specify how patients were selected for inclusion, and with regard to other complications they reported an unusual low number of incidents (1.08% of procedures) (24).

That we had to resolve to referring an excluded article when discussing a major outcome such as mortality illustrated one potential weakness of our approach – we might have applied much rigorous exclusion criteria. However, the informed reader needs to evaluate the external validity of the included studies, and we considered a comprehensive description of the sampling process as imperative for this purpose. We have also chosen to exclude more specific procedures such as bronchoscopy in the ICU, endobronchial ultrasound (EBUS), and interventional ultrasound, which should be topics of separate, future reviews.

Although bronchoscopy appeared to be a safe procedure in terms of mortality, bleeding, and pneumothoraces, it was difficult to conclude regarding the frequency of other specific complications. The inter-study variation in definitions of specific complications was considerable if the outcomes were defined at all. In particular, this could be exemplified by the variation in desaturation and bleeding rates, as well as cough, health care utilisation, and discomfort. The variation in definitions of ‘complications’ can have several reasons, but it is likely due to the researchers’ and clinicians’ perception of what can be considered significant complications, and which adverse events are relevant for a specific patient group. Definitions may also vary due to available tools for recording adverse events. We also observed a lack of studies addressing complications and discomfort related to specific sampling techniques, sedation, duration of the procedure, and experience of the bronchoscopist. Similarly, there were few articles that reported patient characteristics related to safety and discomfort, such as indication for bronchoscopy, comorbidities, age, and pre-procedural anxiety. In the case of hypoxaemia, only two of the studies that provided desaturation rates specified a pre-procedural minimum resting/room air saturation of the participants (>90%) (77, 79). Few subtopics in our article present predictors of complications, and we cannot, finally, conclude on predictors of complication. This is mainly due to predictors not being presented in the reviewed articles, which could result from insufficient statistical power.

## Conclusion

To conclude, bronchoscopy is a safe procedure in terms of complications such as mortality, pneumothorax, and bleeding that necessitate intervention. However, we should be able to inform patients in less broad strokes, with details concerning risk of both complications and what clinicians would characterise as discomforts. To provide this information, we need a sufficiently powered, prospective study on a well-described sample with clear definitions of complications that at least include mortality, pneumothorax, desaturation, bleeding, hypotension, arrhythmia, fever, and ‘willingness to return’. Characteristics of participants and procedures should be related to the outcomes in order to identify high-risk procedures. In addition, all complications should be characterised in terms of necessary intervention.
